# Snakebite epidemiology, outcomes and multi-cluster risk modelling in Eswatini

**DOI:** 10.1371/journal.pntd.0011732

**Published:** 2023-11-10

**Authors:** Sara Padidar, Ara Monadjem, Thea Litschka-Koen, Brent Thomas, Nondusimo Shongwe, Clare Baker, Lindelwa Mmema, Trevor Sithole, James Murray, Nicholas R. Casewell, Jonathan Pons, David G. Lalloo, Robert A. Harrison, Ymkje Stienstra, Wisdom M. Dlamini

**Affiliations:** 1 Department of Biological Sciences, University of Eswatini, Kwaluseni, Eswatini; 2 Eswatini Antivenom Foundation, Simunye, Eswatini; 3 Eswatini Snakebite Research and Intervention Centre, Simunye, Eswatini; 4 Mammal Research Institute, Department of Zoology & Entomology, University of Pretoria, Hatfield, Pretoria, South Africa; 5 Centre for Snakebite Research and Interventions, Liverpool School of Tropical Medicine, Liverpool, United Kingdom; 6 Research Unit, Ministry of Health, Mbabane, Eswatini; 7 University of Groningen, University Medical Center Groningen, Department of Internal Medicine/Infectious Diseases, Groningen, The Netherlands; 8 Department of Geography, Environmental Science and Planning, University of Eswatini, Kwaluseni, Eswatini; University of Peradeniya Faculty of Medicine, SRI LANKA

## Abstract

**Background:**

Halving snakebite morbidity and mortality by 2030 requires countries to develop both prevention and treatment strategies. The paucity of data on the global incidence and severity of snakebite envenoming causes challenges in prioritizing and mobilising resources for snakebite prevention and treatment. In line with the World Health Organisation’s 2019 Snakebite Strategy, this study sought to investigate Eswatini’s snakebite epidemiology and outcomes, and identify the socio-geographical factors associated with snakebite risk.

**Methodology:**

Programmatic data from the Ministry of Health, Government of Eswatini 2019–2021, was used to assess the epidemiology and outcomes of snakebite in Eswatini. We developed a snake species richness map from the occurrence data of all venomous snakes of medical importance in Eswatini that was subjected to niche modelling. We formulated four risk indices using snake species richness, various geospatial datasets and reported snakebites. A multivariate cluster modelling approach using these indices was developed to estimate risk of snakebite and the outcomes of snakebite in Eswatini.

**Principal findings:**

An average of 466 snakebites was recorded annually in Eswatini. Bites were recorded across the entire country and peaked in the evening during summer months. Two cluster risk maps indicated areas of the country with a high probability of snakebite and a high probability of poor snakebite outcomes. The areas with the highest rate of snakebite risk were primarily in the rural and agricultural regions of the country.

**Significance:**

These models can be used to inform better snakebite prevention and treatment measures to enable Eswatini to meet the global goal of reducing snakebite morbidity and mortality by 50% by 2030. The supply chain challenges of antivenom affecting southern Africa and the high rates of snakebite identified in our study highlight the need for improved snakebite prevention and treatment tools that can be employed by health care workers stationed at rural, community clinics.

## Author summary

Snakebite from venomous snakes is a medical emergency requiring immediate critical care by a medical team. Failure to correctly manage a snakebite can lead to permanent disability or even death. The dearth of data on the frequency, location and populations affected by snakebite, as well as the outcomes of snakebite, in many regions of the world means that this tropical disease remains one of the most neglected. We studied snakebite over two years in Eswatini using data on hospitalisations, snake locations, habitat, environmental and socioeconomic factors to develop two nation-wide risk maps: one estimating the probability of snakebite and the other one the probability of poor outcomes in the case of a snakebite. The results of this study can enable national policy makers and health programmes to better plan and allocate resources, as well as implement targeted prevention strategies to meet the global goal of halving deaths and disabilities caused by snakebite envenoming.

## Introduction

In 2017, the World Health Organization (WHO) designated snakebite envenoming a neglected tropical disease [1], which was followed two years later by the launch of the Snakebite Strategy with the aim of halving mortality and morbidity from snakebite by 2030 [2]. The WHO estimates that each year 5 million people are bitten by snakes, with up to 2.7 million people becoming critically ill, 130,000 suffering mortality, and a further 400,000 becoming physically disabled [3]. In sub-Saharan Africa, annual estimates are 1 million snakebites with only 360,000 presenting to health facilities resulting in approximately 20,000 deaths [4,5]. Though robust studies are rare, snakebite incidence estimates have been reported from some African countries including Cameroon, Kenya, Mozambique, Nigeria, Rwanda, Senegal and South Africa [6–10].

Globally, the incidence and severity of snakebite envenoming has been underestimated with the condition receiving little attention [4,6,10,11]. There are a number of challenges in gathering accurate data on incidence and severity, which include poor recording of snakebite cases that present to health facilities, patients’ willingness to come to health facilities and their confidence in the health system to treat snakebite when there is lack of timely access to affordable and effective antivenom treatment, and patient beliefs and/or health seeking behaviour [12–16]. Snakebite victims commonly report self-administering first aid measures, or consulting traditional healers instead of, or prior to, coming to a health facility [11–13,17]. Patients who die before reaching a healthcare facility or who choose to only seek treatment from a traditional healer will not present to the health system and therefore will not be counted in routinely collected national statistics.

Epidemiological studies from Africa and Asia show snakebite envenoming to disproportionally affect rural, farming and impoverished populations [5,18–20]. Children and young adults comprise the majority of the cases [5,7–9,21,22] followed, to a lesser extent, by the elderly [23,24]. In addition, susceptibility to snakebite is increased in situations where individuals live in houses whose construction does not prevent snake ingress and that have limited lighting and sanitation facilities [25] or where people need to collect firewood [8], factors that are underpinned by poverty. Understanding and modelling risk of snakebite may be one strategy to reduce the incidence and improve treatment outcomes of snakebite [26–29]. These studies have used snakebite incidence data, habitat suitability of common venomous snakes, land use land cover, geographic and socioeconomic status of snakebite patients, and/or venomous species distribution and occurrence data to geo-spatially model the risk of snakebite within a particular region of a country.

Eswatini, a landlocked country in southern Africa, has a population of 1.3 million, of which almost 80% live rurally and 30% have a household income of under US$1.90 a day [30]. Despite its small area and population, snakebite is an important and challenging public health issue [13,31,32]. The country is geographically diverse, with four distinct agroecological zones determined by elevation and rainfall [33], creating suitable habitat for over 60 snake species in 39 genera [34,35]. Eleven of these species are venomous and medically important, including seven species that bite frequently and are associated with serious or life-threatening envenoming (category 1). Four species bite frequently but rarely cause serious or life threatening envenoming (category 2), as classified by WHO [36]. The seven category 1 species are the puff adder (*Bitis arietans*), black mamba (*Dendroaspis polylepis*), boomslang (*Dispholidus typus*), rinkhals (*Hemachatus haemachatus*), snouted cobra (*Naja annulifera*), Mozambique spitting cobra (*Naja mossambica*), and vine snake (*Thelotornis capensis*) [31]. The four category 2 species are berg adder (*Bitis atropos*), Bibron’s stiletto snake (*Atractaspsis bibronii*), rhombic night adder (*Causus rhombeatus*), and snouted night adder (*Causus defillippii*).

The number of bites by these medically important snakes in Eswatini has not previously been reported. In conjunction with our other studies assessing antivenom provision for Eswatini [37], the objectives of this study were to: 1) determine the number of snakebites in Eswatini by compiling those reported to health facilities; 2) describe the epidemiology of snakebite in the country and associated factors; and 3) to develop two risk maps: one for the probability of getting a snakebite and another for the probability of a poor outcome for a snakebite victim.

## Methods

### Snakebite data

Anonymised programmatic data from the Neglected Tropical Diseases Programme, Ministry of Health, Eswatini (MOH) snakebite database, was compiled between October 2019 and September 2021. Twenty health facilities have the capacity to treat snakebite in Eswatini. Sixteen health facilities, comprising of government (n = 10), faith based (n = 1), industry (n = 3), NGO (n = 1) and private (n = 1) hospitals and health centres, reported to treat snakebite patients during the reporting period. Snakebite data was reported by emergency rooms, outpatient, and paediatric departments at these health facilities. This database included the passive reporting of short-term adverse events of the polyvalent and monovalent (for use in *Dispholidus typus* bites only) SAIMR snake antivenoms (both manufactured by South African Vaccine Producers (SAVP)).

### Epidemiological data analysis

Descriptive statistics were calculated using sociodemographic variables available from the MOH anonymised programmatic snakebite database included gender, age, and occupation. The International Standard of Occupations Classification [38] was used to group the occupations, with additional categories created to accommodate those reporting to be unemployed, students, self-employed (without an industry identified), retirees, and children under 5 years old too young to be at school. Snakebite variables analysed included date and time of snakebite, anatomical site of the bite, circumstances of bite, clinical presentation and syndrome, type of first aid administered, treatment administered, and outcome of snakebite.

Kruskal-Wallis test [39] with a paired Dunns’ multiple comparison test using the Bonferroni method [40,41] was used to determine the association between sociodemographic variables and snakebite variables. Relative risk ratios of snakebite for gender and age were calculated using 2017 Population and Housing Census data obtained from the Central Statistics Office [42] as the denominator.

Chi-squared test of independence was used to measure association between categorical variables. The Cramér’s statistics (Cramér’s V) was used to interpret these association estimates and this method is often reported in addition to chi-squared tests as an effect size index [43,44]. Cramér’s V index ranges from 0 to +1 and a higher Cramér’s V value indicates a stronger association between variables, whilst a lower one indicates a weaker association [44].

A Generalised Linear Model (GLM), fitted to a negative-binomial distribution with a log link function, was also developed to explore the link between elevation and the incidence of snakebite. The explanatory variables of gender, age, and occupation of the snakebite victims were tested. We used a step-wise selection to manually remove the worst performing variable at each step. To test model fit, we calculated the pseudo R^2^ using the R package “modEvA” [45].

Statistical significance was determined by p values <0.05. All statistical analyses were carried out in ‘R’ version 3.6.2 [46], using packages “MASS” [47], “lsr” [48], and “dunn.test” [49].

### Snakebite spatial risk assessment

#### Data sources

To assess the snakebite risk in Eswatini, we formulated four widely used risk indices using spatial datasets and information about reported snakebites, using the approach employed by Dlamini et al [50] and Szalinkska et al [51]: hazard, exposure, susceptibility, and resource scarcity [52,53]. The last two together constitute what is commonly termed as vulnerability. The datasets used for estimation of these four risks are listed in S1 Table.

The distribution of the 11 medically important venomous snake species in Eswatini were modelled from occurrence records derived from two sources: observational records vouched for by photographs and museum records vouched for by specimens. The observational data came from trained snake rescue volunteers, security firms, members of the public and snake enthusiasts who submitted pictures, locations, and dates of snakes (dead or live) sighted in the country between 2019 and 2021. This data was submitted to Eswatini Antivenom Foundation and managed by University of Eswatini. Each snake was positively identified by a herpetologist, zoologist and/or trained snake enthusiast. The museum records were based on specimens with locality data housed at the Durban Natural Science Museum, Ditsong National Museum of Natural History, both in South Africa, and the Eswatini National Museum of Natural History, Eswatini for these 11 species collected during past surveys [54] as well as the South African reptile atlas [55]. In addition, we sourced distribution records for these 11 snake species in southern Africa based on the Global Biodiversity Information Facility (GBIF) records (www.gbif.org) (downloaded on 24 February 2022).

Socio-economic and demographic national data were derived from the 2017 Population and Housing Census data obtained from the Central Statistics Office [42]. The data is disaggregated at enumeration area (EA) level which is the smallest census unit in the country. The 2326 EAs were the lowest-level appropriate unit of analysis ranging in size from 0.012km^2^ to a maximum of 194.19km^2^, averaging at 7.5km^2^. The large EAs are mainly vast areas of sparsely populated landscapes such as commercial forestry and sugarcane plantation, livestock ranches and protected areas.

### Snakebite hazard mapping

Mapping the snakebite hazard is essential for understanding snakebite risk. We used the locality information for cases reported between October 2019 and August 2021 (n = 821), analysed separately to the rest of the anonymous MOH snakebite database, to geocode the reported incidents and to develop the hazard map. Geocoding of cases was based on the reported localities using an address locator created using the EAs and various points of interest (schools, chiefdoms, etc.). The snakebite incident points were then placed within the matching area name. The map of the snakebite incidents was then used to estimate the proximity of all the reported incidents to the nearest 1%.

A snake richness map was developed from the occurrence data of all the known category 1 and 2 venomous snake species in Eswatini. The species richness map was created by following Monadjem et al [56] to model the distribution of these venomous snake species in Eswatini, using Maxent version 4.1.1 [57,58]. Models were run at a resolution of approximately 5 km (2.5 arc min) using BIOCLIM variables from the WorldClim database [59], as well as altitude [59], altitudinal roughness extracted from altitude using the program DIVA-GIS (available at www.divagis.org), and ecoregions as classified by Olson et al. [60]. Since BIOCLIM variables are frequently strongly correlated, we assessed the correlation between these variables in the R package “usdm” [61], and then removed variables with variance inflation factor (VIF) >10 using the function “vifstep” [62]. This resulted in the inclusion of 11 variables (altitude, altitudinal roughness, Ecoregions, Bio2, Bio3, Bio8, Bio9, Bio13, Bio14, Bio15, Bio19). We ran Maxent models in R version 3.6.2 [46] using the package “dismo” [63]. We used hinged and categorical variables that smooth variable responses and generally improve model performance [64,65]. We converted the predicted model outputs from Maxent (probabilities of suitability) into “presence-absence” maps using species-specific thresholds that maximized the sum of sensitivity and specificity, which is appropriate for presence-only data [66]. We summed the modelled distributions of all the snakes to quantify species richness using the “Raster Calculator” in QGIS [67].

Hazard risk was assessed as a function of snake species richness and the frequency of reported snakebite cases in a given EA. Therefore, EAs considered as high hazard snakebite areas would be those with the high frequency of reported snakebite cases and relatively higher diversity of venomous snake species (species richness). Similar hazard analysis approaches have been applied in health and other fields [51,68]

### Snakebite exposure mapping

A review of snakebites reported to the Eswatini health system between 2019–2021 (see “Snakebite data” above for details) was performed to ascertain the factors that may expose humans to snakebite in Eswatini. We defined exposure factors as spatially explicit environmental variables that are thought to be associated with situations that increase human-snake contact and may thus be important for exposing humans to snakebite. These exposure factors were the proportion of trees, proportion of cropland and proportion of built-up areas, building density, building occupancy, degree of fragmentation (e.g., landcover edge density and landcover Shannon diversity) and the presence of poultry (S1 Table).

### Snakebite susceptibility mapping

We defined susceptibility as behavioural factors linked to demographic and socio-economic factors associated with snakebite [69]. Susceptibility increases in situations where individuals live in houses with construction that do not prevent snake ingress, that have limited lighting and sanitation facilities, and in cases where people are collecting firewood. These housing construction, lighting, cooking fuel and sanitation factors are underpinned by poverty. The MOH snakebite database used in this study identified that children and young adults generally comprise most of the cases in Eswatini followed, to a lesser extent, by the elderly. Consequently, susceptibility was estimated using the proportion of elderly people, proportion of children and youth, poverty headcount, proportion of households that use firewood, candles and paraffin for lighting and/or cooking. The poverty incidence is the proportion of the population that live below the poverty line of less than USD 2 a day [70].

### Access to health care (Resource scarcity)

A major concern regarding snakebite envenoming is that treatment requires specialized training and healthcare resources. Consequently, the areas at highest risk of experiencing healthcare resource scarcity in the country need to be determined. Access to healthcare facilities was calculated as the estimated travel time to the nearest healthcare facility that has the required equipment, treatment, and personnel trained on snakebite treatment. Notably, not all health facilities in Eswatini have the necessary equipment nor the personnel trained to respond to snakebite emergencies. Twenty health care facilities with the capacity to administer antivenom were used in this analysis, comprising of government (n = 10), faith-based (n = 1), non-profit (n = 1), industry (n = 3) and private (n = 5) health centres and hospitals. For this analysis, we used the same 16 health facilities who reported snakebites during 2019–2021, plus four private health facilities.

The travel time for a patient to the nearest health facility was estimated using the methodology described by Weiss et al., [71]. The approach makes use of detailed Open Street Map and Google transportation network data via the Google Earth Engine [72] to quantify travel time to the health facilities at a spatial resolution of approximately 1km^2^. The method estimates time of travel for individuals with and without access to motorised transportation. In addition to the roads, the technique considers maps of land features such as rivers and dams, the type of land cover, and the slope of the land to allocate an estimated speed of travel to every pixel. This includes the type of road (if present), motorised and unmotorised maps including walking only, and accounts for international borders and elevation in estimating the travel speed.

### Snakebite risk analysis

We created a hypothetical polygon containing the worst-case values for all the variables used in each risk index. This polygon was used as a reference for ranking the relative risk of snakebites in the country. The similarity search technique was used for this purpose. This technique identifies those areas, in this case EAs, that are most similar (or most dissimilar) to the variables in the reference EA which has the highest possible values. Where there is more than one contributing variable to match, similarity is based on averages for each of the variables of interest. During the process, all the variables are first standardized using a z-transform where the mean for all values is subtracted from each value and divided by the standard deviation for all values. The standardization process allows for comparison of scores from different distributions such as ratios, distances and counts [73]. The vectors of standardized data for each EA (a candidate feature) and the vectors of standardized data for the hypothetical worst-case scenario (a target feature) were then compared using a cosine similarity. The cosine similarity of two vectors, *A* and *B*, was computed as follows:

$$Cosine\;similarity\;index=\frac{\sum_{i=1}^{n}A_{i}B_{i}}{\sqrt{\sum_{i=1}^{n}{A_{i}}^{2}\sqrt{\sum_{i=1}^{n}{B_{i}}^{2}}}},$$

The output from this analysis contains the input variables being matched along with all of the matching EAs ordered by similarity. After processing all the EAs, they were then ranked from smallest similarity index (most similar) to the largest (most dissimilar to the worst-case target polygon). Ultimately, all the EAs were ranked from 1 (very similar to the worst-case feature) to 2036 (very dissimilar to the worst-case feature). The EAs were re-ordered in such a way that the EAs that closely match the reference worst case EA had the highest index values for all the risk factors. These values were then normalized to a range from 0 to 1. The spatial risk analysis was conducted in ArcGIS Pro 2.9.3 [74].

### Risk profiling

To develop strategies for the prevention and control of snakebites, information combining all the risk indices is required. A map that characterizes the geographic variations in risk profiles is therefore imperative. For this purpose, a multivariate clustering technique was used to partition the country into zones with similar snakebite risk profiles using the four risk indices as inputs. The multivariate clustering approach utilizes unsupervised machine learning methods which searches for natural clusters in the data. This method has been previously used to assess risk for other health conditions [50,75].

The EAs with the highest hazard, exposure, susceptibility, and healthcare scarcity rankings were allocated a seed value of 1 and all others a value of 0. The seed value represents a number used to initialize a random number generator and specifies a stream from a set of possible random numbers. This clustering approach seeks a solution where all the features within each cluster are as similar as possible, and all the clusters are as dissimilar as possible. This seeding approach is efficient and ensures that the exact same result is obtained every time the analysis is undertaken. The assessment of feature similarity was based on the different identified risk levels whilst the clusters were created using the k-means algorithm [76]. The k-medoids algorithm segregates features so that the differences among the features in a cluster, over all clusters, are minimized. It has the advantage of being more robust to noise and outliers in the input features [77]. Since the algorithm is NP-hard, a greedy heuristic is used to cluster features. The clustering effectiveness is measured using the Calinski-Harabasz pseudo F-statistic [78], which is a ratio of between-cluster variance to within-cluster variance calculated as follows [73]:

$$\frac{\left(\frac{R^{2}}{n_{c}-1}\right)}{\left(\frac{1-R^{2}}{n-n_{c}}\right)}$$

where:

$$R^{2}=\frac{\sum_{i=1}^{n_{c}}\sum_{j=1}^{n_{i}}\sum_{k=1}^{n_{v}}{(V_{ij}^{k}-\bar{V^{k})}}^{2}}{{\sum_{i=1}^{n_{c}}\sum_{j=1}^{n_{i}}\sum_{k=1}^{n_{v}}(V_{ij}^{k}-\bar{V_{t}^{k})}}^{2}}$$

*n* = number of features

*n*_*i*_ = the number of features in cluster i

*n*_*c*_ = the number of classes (clusters)

*n*_*v*_ = the number of variables used to cluster features

$V_{ij}^{k}$ = the number of the *k*^*th*^ variable of the *j*^*th*^ feature in the *i*^*th*^ cluster

*V^k^* = the mean value of the *k*^*th*^ variable

$V_{t}^{k}$ = the mean value of the *k*^*th*^ variable in cluster *i*

The R^2^ value is computed for each risk factor and reflects how much of the variation in the data is explained after the clustering process.

The cluster analysis was performed in two ways; one analysis where healthcare resource scarcity was not included (i.e., only focusing on the risk of anyone being bitten) and another where the healthcare resource scarcity was included. The latter provided an indicator of the probable outcome of snakebite and provides a basis for recommendations for resource allocation, particularly the supply of antivenom and/or capacity building of health facilities and medical personnel. The risk clusters were then presented in the form of maps and accompanying box plots showing the variations in in the risk factors. Google Earth vs 9.171.0.0 was used to identify the localities and characteristics of EAs in the risk clusters.

## Results

**Fig 1:**
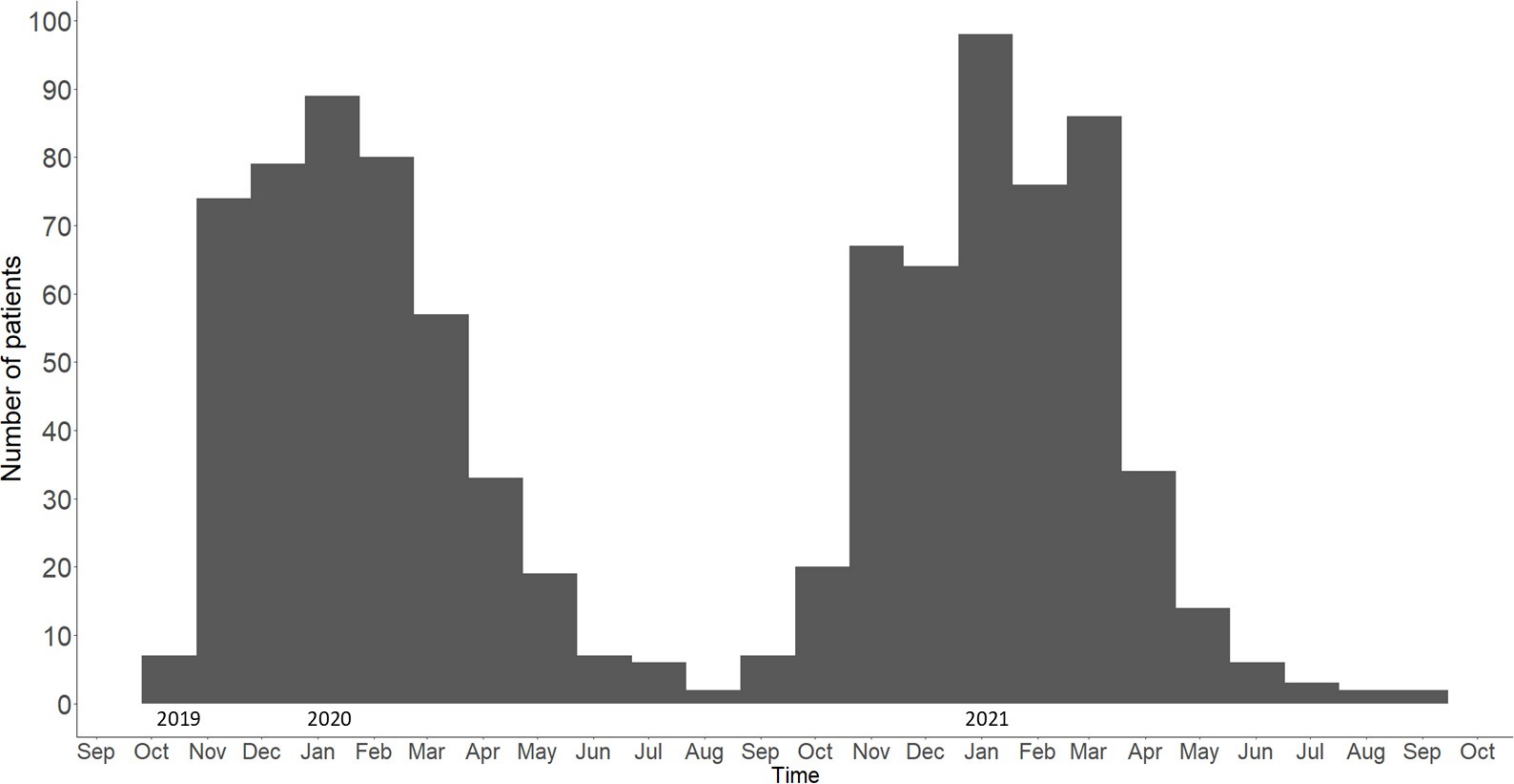
Frequency of snakebite patients presenting to health facilities in Eswatini between October 2019 to September 2021.

A total of 932 snakebites were recorded between October 2019 and September 2021. Most patients were bitten during the summer months between November to March each year, peaking in January (Fig 1). The average rainfall was 126 mm (71–253 mm), and mean minimum and maximum temperatures were 17 ⁰C (14 ⁰C– 19 ⁰C) and 27 ⁰C (24 ⁰C– 32 ⁰C) respectively during the peak biting months (S1 Fig). Patients reported being bitten throughout the 24-hour period, with 30% of snakebite patients reporting being bitten between 18:00 and 22:00 (S2 Fig).

Over 35% of patients were asymptomatic when they presented to the health facility. Just over half (55%) of bites were cytotoxic causing either mild swelling, painful progressive swelling or venom ophthalmia. Neurotoxic bites accounted for less than 5% of all bites and only two patients (0.2%) were recorded to present with bleeding syndrome. Bleeding syndrome is identified using the 20-minute whole blood clotting test [79], which should be carried out on all patients presenting with snakebite as part of the Eswatini national snakebite management guidelines [80]. The result of this test was not available in the snakebite patient database.

**Fig 2:**
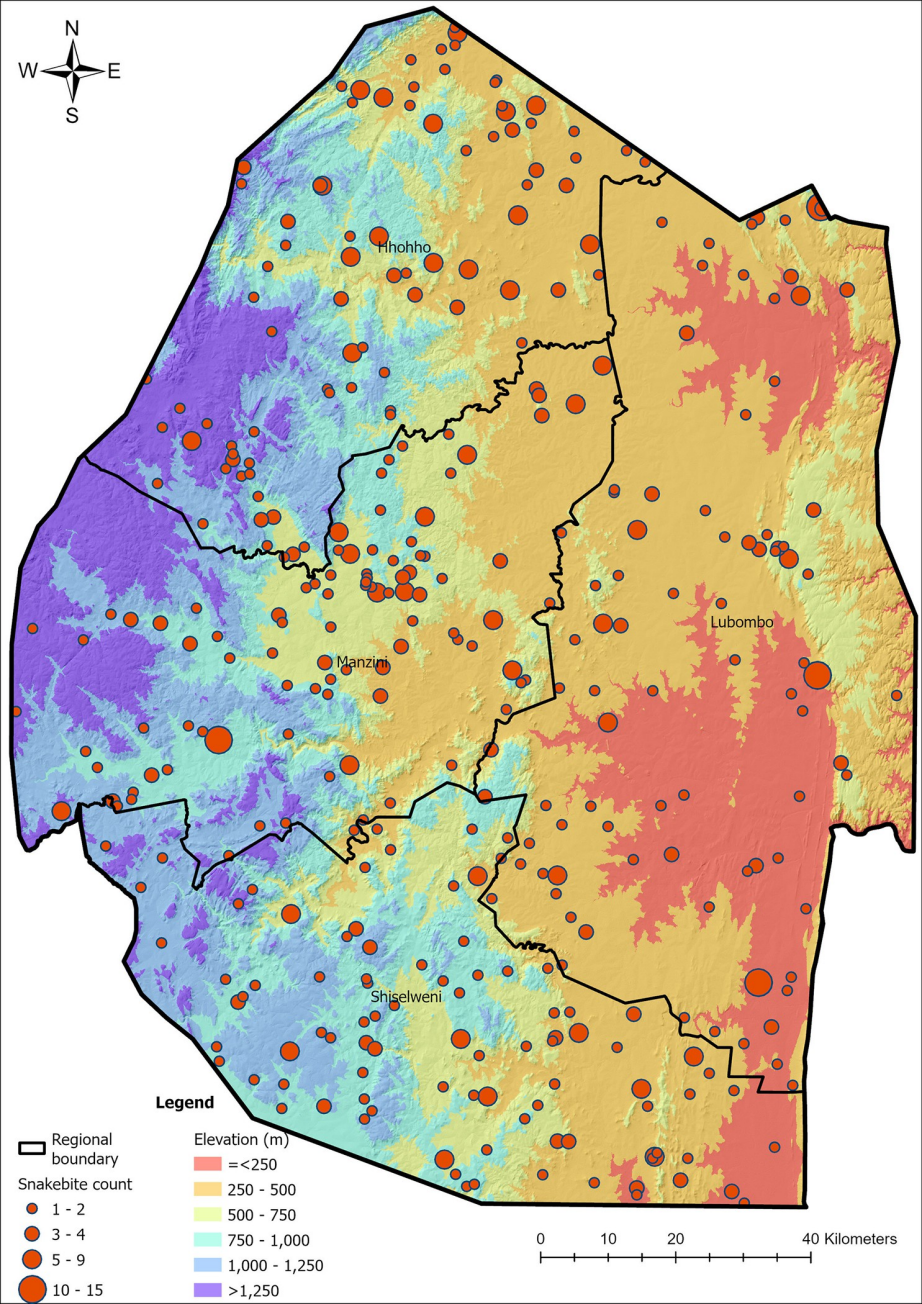
Incidence map of snakebites in Eswatini between October 2019 to September 2021. Made using shape files from the Eswatini Central Statistics Office (with permission).

Geocoding snakebite localities revealed bites occurred across all elevations, ranging between 71 m to 1499 m (Fig 2). Generalised linear modelling with negative binomial demonstrated elevation to significantly explain snakebite incidence (p = 0.0137, pseudo R^2^ = 0.03), with 3.8% (SE = ±1.4%) decrease in snakebite incidence for every 100 m gained in elevation. None of the other variables (gender, age, and occupation of the snakebite victims) in the model were significant. There were no associations between the seasons (spring, summer and autumn) and elevation. However, during the dry winter months of June to August, twenty-two patients presented with snakebite, with fifteen of these patients bitten at low elevations (below 500 m) and only one patient reported to be bitten at an elevation above 1000 m.

### Sociodemographic characteristics of snakebite patients

More male than female snakebite patients were reported at the 16 health facilities, with the relative risk for males to females calculated as 1.29 (95% CI = 1.13–1.46; Table 1). Age of snakebite patients ranged from 0–100 years, with 55% of bites occurring in those under the age of 30 years, which reflects the population [42]. The age group that presented with snakebite to health facilities the most was 10–19 years (27%; Table 1). The relative risk for children aged 0–9 years was 0.51 (95% CI 0.42–0.62) and for 10–19-year-olds was 1.46 (95% CI 1.26–1.69).

**Table 1:** Sociodemographic overview of snakebite patients in Eswatini.

Parameter	No. of patients (%)	Relative Risk (95% CI)
**Sex**		
Male	511 (55)	1.29 (1.13–1.46)
Female	421 (45)	
**Age (years)**		
0–9	116 (12.5)	0.51 (0.42–0.62)
10–19	252 (27.0)	1.46 (1.26–1.69)
20–29	174 (18.7)	1.14 (0.96–1.35)
30–39	135 (14.5)	1.68 (0.97–1.40)
40–49	65 (6.9)	0.92 (0.71–1.19)
50–59	45 (4.8)	0.96 (0.71–1.30)
60–69	23 (2.5)	0.73 (0.44–1.11)
70–79	17 (1.8)	0.98 (0.61–1.59)
80+	12 (1.3)	1.73 (0.98–3.06)
Not recorded	93 (10.0)	-
**Occupation (ISCO-O8)**		
Managers	0 (0)	
Professionals	7 (0.8)	
Technicians and Associate Professionals	2 (0.2)	
Clerical Workers	1 (0.1)	
Services and Sales Workers	11 (1.2)	
Skilled Agricultural, Forestry and Fishery Workers	20 (2.2)	
Craft and related trades workers	14 (1.5)	
Plant and Machine Operators and Assemblers	2 (0.2)	
Elementary Occupations	65 (7.0)	
Armed Forces Occupation	4 (0.4)	
Unemployed	212 (23.0)	
Students	327 (35.0)	
Self-employed	13 (1.4)	
Retirees	33 (3.5)	
Children too young to be at school	53 (5.7)	
Unknown	168 (18.0)	

Most snakebite patients (35%) were students (ages 6 years and above), followed by those unemployed (23%), in elementary low paid occupations such as unskilled labourers and domestic workers (7%) and retirees (6%) (Table 1).

### Variables associated with snakebite susceptibility

**Fig 3:**
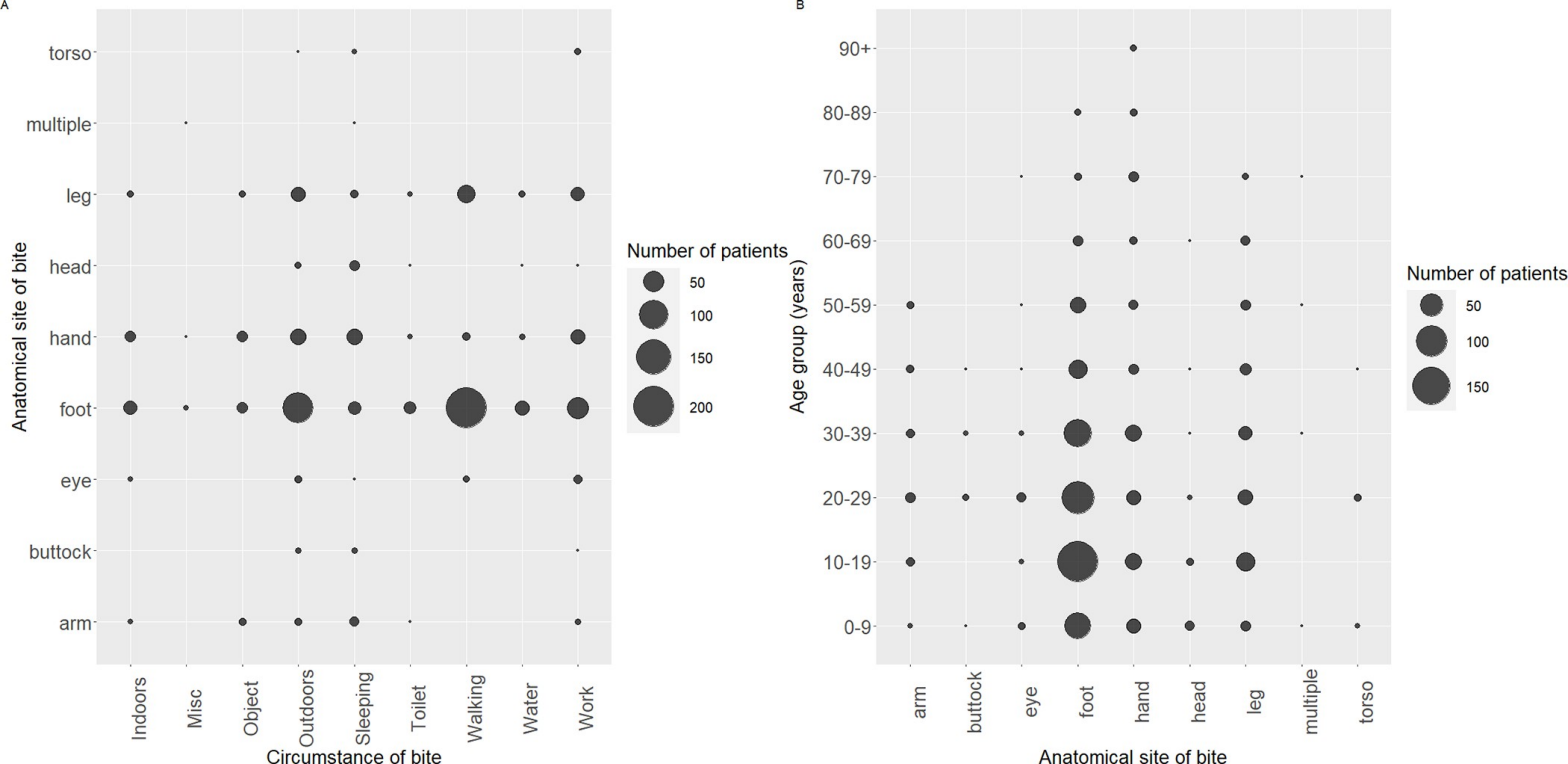
(A) Body part bitten by snake by activity of the person at the time of bite, in Eswatini; (B) Body part bitten by snake by age group of snakebite patient, in Eswatini.

Most bites (65%) occurred outdoors, 13% were indoors, and the location of over 20% of participants was not recorded. Over half (60%) of all bites were to the lower extremities of the foot, ankle, heel or toe, whilst 14% were to the hand, finger or wrist. The position of the bite was significantly and moderately strongly associated with the bite circumstances (χ^2^ = 327.64, d.f. = 64, p-value < 0.001; Cramer’s V = 0.24). Analysis of the activities undertaken when the bite occurred revealed 27% of snakebite patients were walking, with bites to the lower extremities associated with walking, fetching water or being outdoors (Fig 3A). Age was significantly but weakly associated with the position of the bite on the body (χ^2^ = 59.02, d.f. = 24, p-value < 0.001; Cramer’s V = 0.15). Bites to the leg or foot were associated with younger patients, whilst bites to the arm or hand were associated with older patients (Fig 3B). Gender was not significantly associated with anatomical site of snakebite.

### Snakebite management

Prior to arrival at a health facility, 66% of patients had self-administered or received some form of first aid by a friend, relative or traditional healer. One or more first aid measures were implemented, with 83% of these patients presenting with a tourniquet tied close to the anatomical site of the bite. Other measures included making incisions, applying and/or ingesting herbal remedies, or applying a bandage (S2 Table).

The polyvalent and monovalent (for use in *Dispholidus typus* bites only) SAIMR snake antivenoms (SAVP) are registered for use in Eswatini. Over 75% (n = 709) of reported snakebite cases did not receive antivenom. Of these patients, 105 patients (15%) were diagnosed with a snakebite clinical syndrome that would normally warrant the use of antivenom (painful progressive swelling, progressive weakness or bleeding syndrome) [80]. Four of these patients died and one was identified by the treating physician to be left with long term physical impairment after a fasciotomy. Reasons for not administering antivenom in these patients was not indicated. Of the 200 patients that received antivenom (mean = 5 vials, range 1–25 vials), all received the SAIMR polyvalent antivenom, and 16.5% presented with neurotoxic envenoming, 77.5% with signs of possible cytotoxic envenoming (painful progressive swelling or mild swelling), 3.5% with no symptoms, 2% patients with unknown clinical presentation and one patient was recorded to have received 4 vials of antivenom for venom ophthalmia.

Adverse reactions to antivenom were recorded in 35% of the 200 patients who received SAIMR polyvalent antivenom, with the most common reaction recorded to be urticaria (S3 Fig). Severe adverse reactions leading to discontinuation of antivenom treatment were reported in four patients, of which two patients received adrenaline prior to receiving antivenom. Subcutaneous adrenaline was administered prior to giving antivenom in 167 (84%) of the 200 patients. It is noteworthy that of these 167 patients, 39% reported experiencing adverse reactions to the SAIMR polyvalent antivenom. Of the remaining 33 patients that did not receive adrenaline prior to antivenom administration, 15% experienced adverse reactions.

### Treatment outcomes of snakebites

Out of the 932 patients that presented with snakebite, 93% made a full recovery. Ten patients (1%) died, and 8 patients were left with long term physical impairment. No information was recorded regarding the outcome of 5% of patients. The two patients that presented with bleeding syndrome did not receive any antivenom and made a full recovery. Of the 10 patients that died, 5 of them received between 2–12 vials of antivenom, four patients did not receive antivenom and either died en route to hospital or in hospital. There was no information regarding the antivenom administration to the last patient that died from snakebite. The five patients that received antivenom were all admitted to the general medical inpatient ward. Four of the deceased patients were aged between 1–6 years, 2 patients aged 11–19, and 3 patients were aged 37–53 years, and 1 patient was over 70 years old. Eight of the patients who died presented with progressive weakness associated with a neurotoxic bite, and two patients presented with a painful progressive swelling associated with a cytotoxic bite to the face or head. Both patients who died from cytotoxic bites were male children (6 years or less) who were bitten whilst sleeping indoors and their treatment was delayed because they both first arrived at a health facility that did not have any antivenom. The first child arrived at a health facility with a tourniquet around his head, then later died in an ambulance en route to the second health facility. The second child died after having received CPR then 2 vials of antivenom at the second health facility. No adverse reactions to the antivenom were reported.

Seven of the eight snakebite survivors who were left with long term physical impairment, as assessed by the treating physician at the time of discharge, had presented with mild swelling or painful progressive swelling. During their snakebite treatment, these eight patients underwent one or more of the following procedures leaving them with long term physical impairment as assessed by the treating physician: skin grafts (n = 3), fasciotomy (n = 3), and/or amputation (n = 3). One patient, recorded to present with neurotoxic envenoming, also underwent a fasciotomy resulting in permanent scarring and physical impairment.

### Assessing snakebite risk

#### Hazard risk

**Fig 4:**
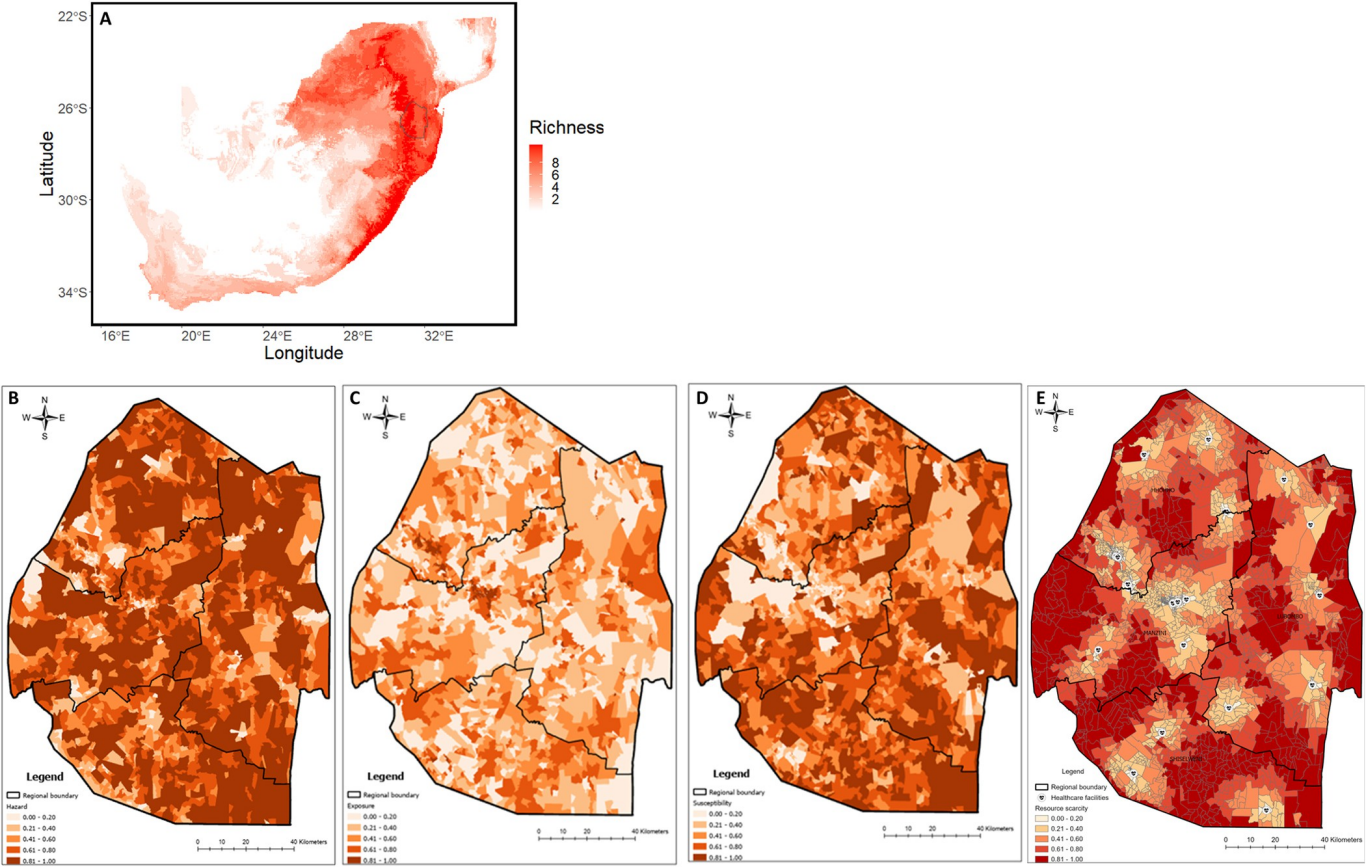
Species richness of the eleven venomous snakes (considered in this study) in Southern Africa (A), and the hazard (B), exposure (C), susceptibility (D) and health facility access scarcity (E) risk maps associated with snakebite in Eswatini. For maps B-D, the darker the area the greater the risk. For map E, the darker the area the scarcer the access to health facilities. Map A made with Natural Earth. Free vector and raster map data @naturalearthdata.com. Maps B-E made with shape files from the Eswatini Central Statistics Office (with permission).

Species richness of the eleven venomous species considered in this study peaks in the eastern parts of South Africa, Eswatini and neighbouring parts of Mozambique (Fig 4A), with only minor variation in species richness across Eswatini. Based on the modelled distribution of these species, any location in Eswatini harbours between 8–11 species of venomous snakes (S4 Fig). The modelled snakebite hazard (Fig 4B) indicates that areas with the lowest hazard risk correspond to urban areas, protected and conservation areas where human populations are low, as well as areas bordering South Africa in the west and southwest of Eswatini, and those bordering Mozambique in the east. Considering the relatively uniform species richness across the country, the areas with elevated snakebite hazard correspond to populated areas in both rural and peri urban areas.

#### Exposure risk

The snakebite exposure risk was not uniform throughout the country, with the highest risk of exposure (Fig 4C) associated with densely populated and built-up areas. High resolution images of these high-risk areas indicate these to be peri urban areas close to tar roads and towns, with a mixture of land uses ranging from homesteads on small plots that had livestock and some crops to industrial estates. Lowest risk areas are characterized by very low human population density such as protected and conservation areas as well as commercial farmlands.

#### Susceptibility risk

Susceptibility shows marked contrasts particularly between rural and urban environments. Notably, the southern administrative region of Shiselweni had the highest susceptibility (Fig 4D). Similarly, most rural areas exhibit high susceptibility to snakebites. High resolution imagery of these areas indicates that these high-risk areas correspond to rural communities that are typically situated away from towns and accessible mainly by unpaved roads. Such areas are also characterized by subsistence farming, higher rates of unemployment and poverty, and most use firewood for cooking, rely on the river or a communal tap for water in addition to typically having poor sanitation facilities such as outside pit latrines.

#### Access to health care (Resource scarcity)

Health facilities with the capacity to treat snakebite are distributed across the country (Fig 4E), however their accessibility, and consequently resource scarcity was unevenly distributed. Of the 20 facilities that can treat snakebites, most are concentrated within or in close proximity to urban areas. Where two or more facilities were situated next to or close by each other (i.e., in south of Hhohho region and east of Manzini region), this was reflective of private health facilities being predominantly located close to government health facilities, which invariably were in urban areas. As such, areas adjacent to health facilities with the capacity to treat snakebite had the lowest resource scarcity ranking.

Health facilities in northeast of Hhohho region, east of Manzini region and east-central Shiselweni region had very low resource scarcity values because of increased access and smaller distances to the nearest health facility. Such facilities and their surrounding low risk areas predominantly are typically accessed via paved roads. Areas west of the Manzini region; central, east and south of the Lubombo region; and large areas of the Shiselweni region have high resource scarcity.

#### Snakebite cluster risk mapping

**Fig 5:**
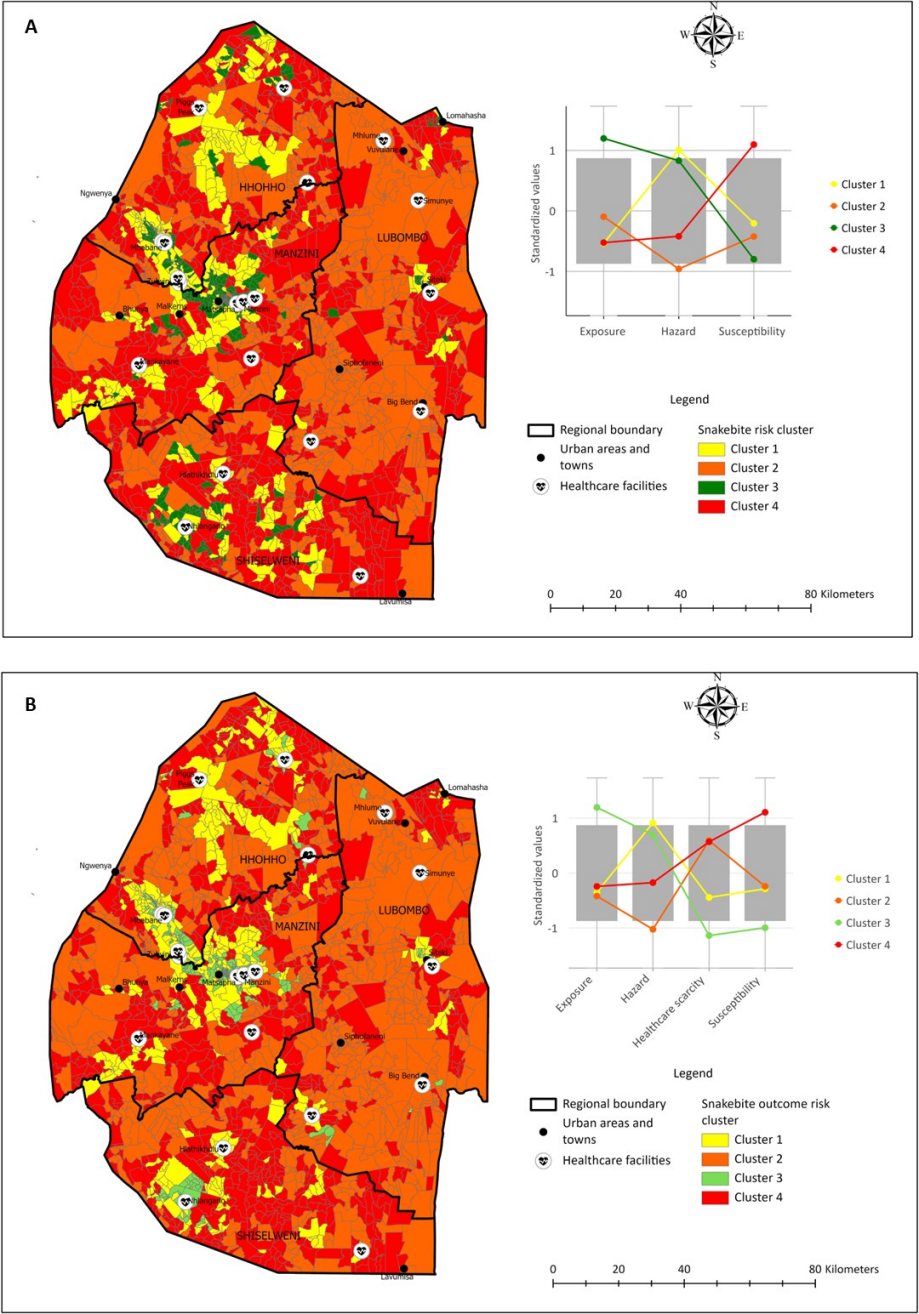
Snakebite risk cluster risk map (A), and snakebite outcome risk cluster map (B). Made with shape files from the Eswatini Central Statistics Office (with [permission).

Cluster analysis (Fig 5A) revealed four clusters highlighting varying degrees of snakebite hazard, exposure, and susceptibility. The risk maps produced in this analysis show areas that have low (0) to high similarity (1) with areas with the worst-case scenario (highest number of reported snakebites). For instance, areas with high snakebite susceptibility are very similar in relation to the factors influencing snakebite, i.e. they have high levels of poverty, high proportion of households that use firewood for cooking, and high proportion of households that use candles for lighting, among other factors.

Cluster 1 (indicated in yellow, Fig 5A) is characterized by low exposure but high snakebite hazard and moderate susceptibility, resulting in a moderate risk of snakebite. EAs in this cluster is dominated by peri urban areas and other high population density areas. Cluster 2 (indicated in orange, Fig 5A) is comprised of EAs that have moderate snakebite exposure and susceptibility but very low snakebite hazard, resulting in a moderate to high risk of being bitten. Cluster 3 (indicated in green, Fig 5A) is comprised of EAs that have very high snakebite exposure and high hazard, but very low susceptibility, resulting in a very low risk of being bitten. Using high-resolution satellite imagery, these EAs were mostly associated with densely populated urban areas. Building density, building occupation density, and the presence of multiple land uses are the exposure variables that push the exposure risk score up. Snake sightings in these areas, as well as snakebite localities recorded as the nearest town would have also increased the reported hazard risk for this cluster. However, the lower levels of poverty, and better access to solid cement or brick buildings, indoor lavatories, high percentage of inhabitants using electricity for lighting and cooking, resulted in very low susceptibility of snakebite, and consequently the low risk of snakebite.

Cluster 4 (indicated in red, Fig 5A) contains EAs that have low snakebite exposure and hazard but very high susceptibility. Enumeration areas in this cluster were at the greatest risk of snakebite. Using high-resolution satellite imagery, these areas were typically associated with rural areas with poor socio-economic conditions and high rates of poverty.

#### Snakebite outcome cluster risk mapping

In total, four (4) clusters emerged as before, with differences in access to healthcare. Availability and fast access to healthcare was moderately scarce in cluster 1, high in clusters 2 and 4, and plentiful in cluster 3.

Densely populated peri urban areas were identified to be where a person is most at risk of being bitten by a venomous snake *and* have a poor outcome (Cluster 4, indicated in red, Fig 5B). Approximately 50% of EAs in the southern Shiselweni region were identified to be at the highest risk of being bitten by a snake and encountering a poor outcome due to scarcity of fast access to health facilities with the capacity to treat snakebite, and high susceptibility to snakebite. High rates of poverty, outdoor pit latrines, and a high percentage of inhabitants using firewood or paraffin for cooking and lighting accounts for the high susceptibility score in this cluster. High-resolution satellite imagery shows EAs in clusters 2 and 4 in this region to be accessible by unpaved road resulting in delayed access to health facilities where life and limb saving treatment can be accessed. EAs in Lubombo region, in the east of Eswatini, were primarily in cluster 2. Whilst access to health resources is challenging in this region, the large areas of commercial agriculture such as sugarcane plantations, as well as protected and conservation areas, lower venomous species richness. Low population density drove down the cluster risk for snakebite outcomes in this region. Both Manzini and Hhohho regions had a diverse mix of clusters with about 25% of EAs in each region identified to be at low and moderate risk of poor snakebite outcomes (clusters 1 and 3, Fig 5B). These EAs were largely the same as those identified in the snakebite cluster risk map (Fig 5A). High resolution imagery confirmed a highway linking EAs in clusters 1 and 3 that traverse the regional boundary, indicating good access to health facilities associated with urban areas. Health facilities not located in urban areas, such as those in the southeast and southwest of Manzini region, and northeast of Manzini region bordering Hhohho region, whilst accessible by tar road, have populations typically living in surrounding area that are only accessible by unpaved road, thereby delaying access to snakebite treatment and increasing risk of poor snakebite outcomes.

## Discussion

Understanding snakebite burden, epidemiology and risks enables governments and health agencies to make informed decisions when allocating resources as well as designing and implementing treatment and prevention measures to achieve the global goal of reducing morbidity and mortality from snakebite by 50% [2]. Despite the reported widespread incidence of snakebites in Eswatini, this is the first analysis through modelling which also identifies geographical locations that are at various levels of snakebite risk and at risk of poor outcomes from snakebite.

The seasonal distribution of snakebites followed weather patterns in Eswatini, with October to March being the ‘wet’ season characterised by rainfall and warmer temperatures, similar to other countries [6,7,22,26,81–84]. As shown in other studies [82], patients were mostly bitten at low geographical elevations where, relative to the rest of the country, temperatures are warmer. Historical trends have shown annual average temperatures have increased by 3⁰C between 1961–2020, and climate change is expected to see mean annual temperatures rise by a further 2⁰C by 2069 [85]. Consequently, this is expected to affect the distribution of biodiversity and ecosystems in Eswatini, including species movements leading to an increase in human-wildlife interactions, including snakebite [86].

Patients aged 10–19 years, and males had a greater relative risk of being bitten than other patients, supporting snakebite data from other countries [7,9,22,26,81–84,87,88]. Targeted public health prevention interventions to these populations through schools, community structures, peer educators should be prioritised, using traditional and social media [31,89]. Most bites were to the foot and 25% of patients were bitten whilst walking, as also reported in other countries [6–9,21,24,87,88]. The peak time of day for snakebites to occur was in the evening and early night between 6-10pm, when most people would be fetching firewood and/or water, preparing evening meals, or securing the homestead before sleep, reflecting findings from other countries [8,22,83,87]. Rural health clinics are normally closed during this time, further challenging timely snakebite treatment in high risk rural and peri urban areas poorly accessible by unpaved roads. Until November 2022, snakebite data was not collected by the national health programme from rural clinics because these clinics do not have the human resources nor the capacity to treat snakebite. Health centres and hospitals typically function with a smaller sized night shift team. Adapting medical team compositions to ensure snakebite expertise is available during the evening and night could be one strategy to reduce the morbidity and mortality associated with snakebite. Incorrect and/or inappropriate application of a tourniquet leads to swelling, confusion of snakebite clinical presentation, and for cytotoxic envenoming cause local sequestering of the venom and accelerated tissue necrosis [90,91]. Application of herbal remedies particularly to broken skin increases the risk of superficial and systemic infections [91]. This study found two thirds of patients had administered some sort of first aid measure, with tourniquets being the most common. Therefore, public programmes promoting more effective snakebite first aid management need to be prioritised particularly in high risk and poor outcome areas. To date, snake safety and snakebite prevention and first aid in Eswatini has been spearheaded by the Eswatini Antivenom Foundation. Interventions employed have included volunteer snake rescuers who remove snakes found in homes and workplaces, public snake-handling trainings, freely available snake identification information, and community and school outreach teaching snake safety and first aid [92]. In collaboration with the Ministry of Health and other national organisations, further interventions are planned including measuring their impact.

Snakebite envenoming is frequently a medical emergency requiring immediate critical care by a medical team. Eswatini has developed national snakebite treatment guidelines [80], and antivenom is listed as a vital medicine to be distributed to all health centres and hospitals through vertical national programmes in its essential medicines list. This enables available antivenom to be prioritised for procurement by the Ministry of Health [93]. Intravenous administration of antivenom therapy neutralizes venom toxins circulating in the patient’s system and is the cornerstone of medical management of snakebite envenoming [94]. However due to critical shortages of SAIMR polyvalent antivenom affecting the southern African region, the procurement of antivenom in Eswatini has been very challenging in recent years. This study found a quarter of all patients presenting with snakebite received SAIMR polyvalent antivenom. Further training, at all levels of the health system, in the clinical uptake of the national snakebite guidelines [80] and support for rational antivenom use is indicated as antivenom was reported to be administered inappropriately to patients presenting with no symptoms of snakebite and to one patient who presented with venom ophthalmia, whilst not given to patients that did need it. Short term adverse reactions to antivenom were reported in 35% of patients, and this percentage has been observed in other studies [22,83,84,95,96]. Subcutaneous adrenaline is used prior to administration of antivenom to mitigate severe adverse reactions [97,98]. However, this study showed 39% of those who received both adrenaline pretreatment and antivenom experienced an adverse reaction, with two patients experiencing severe reactions necessitating discontinuation of antivenom treatment. Further investigations are warranted to elucidate the severity, and the short- and long-term effects of antivenom use. The data also calls into question the rationale used by clinicians for performing fasciotomy, a procedure which is only indicated for the rare event of vascular compromise due to compartment syndrome.

Whilst it was not possible to definitively identify the snakes responsible for the bites, over half of bites presented with syndromes suggesting cytotoxic envenoming (mild swelling or painful progressive swelling), less than 5% of bites presented with progressive weakness associated with neurotoxic envenoming, and 37% presented with no symptoms suggestive of a bite from a non-venomous snake or a dry bite from a venomous snake. Antivenom usage reflects this pattern, with less than 25% of bites receiving antivenom and less than 30% presenting with clinical symptoms necessitating need for antivenom. Furthermore, the frequency of cytotoxic envenoming reflects the venomous snake species in Eswatini with six out of the eleven venomous snake species inducing swelling. These six species are Bibron’s stiletto snake (*Atractaspsis bibronii*), puff adder (*Bitis arietans*), snouted night adder (*Causus defillippii*), rhombic night adder (*Causus rhombeatus*), rinkhals (*Hemachatus haemachatus*), and Mozambique spitting cobra (*Naja mossambica*). Additionally, mildly venomous snakes that do not require medical attention such as the herald snake (*Crotaphopeltis hotamboeia*) and the olive grass snake (*Psammophis mossambicus*) that are common across Eswatini and can also cause mild swelling. Other snakebite studies from South Africa and Sri Lanka have found cytotoxic envenoming to dominate clinical presentation [22,82]. The frequency of cytotoxic envenoming could be explained by potential biases in the patient population presenting to health facilities for treatment. Given the rapid onset of neurotoxic envenoming, such patients may die en route to a health centre / hospital, or at home because relatives take the patient home rather than to the health facility, as reported elsewhere [10]. Consequently, the victim is not included in the health system records. Community surveys will facilitate understanding the extent and reasons for underreporting of snakebite, and the common species involved [10,99,100].

For a snakebite incident to occur, there needs to be a snake-human interaction [101]. Previous studies that have attempted to uncover the incidence of snakebite using various approaches have generally used snake species richness or occurrence data as a key determinant of snakebite risk, with varying degrees of accuracy [26–28]. In contrast, our data, shows that in the small geographical area of Eswatini, species richness alone does not explain snakebite incidence. Snake species richness only indicates the number of snake species that occur or potentially occur in a given area. However, if such species are in low abundance, then the risk of being bitten diminishes. Unfortunately, abundance data for snakes are not available in Eswatini, as is the case for most of the world, which makes it impossible to adequately characterize the true probability of encountering a snake in Eswatini. Nevertheless, the findings in this study show that the exposure, vulnerability and susceptibility to snakebites varies geographically. As it is not yet possible to differentiate between dry bites from venomous species and non-venomous snakebites, the risk modelling of snakebite used distribution of venomous snakes and all snakebite data.

While most of our findings on the geography of bites are in agreement with the current literature [20,26,28,82], subtle patterns and/or clusters emerged. The cluster analysis revealed areas with homogenous levels of exposure, susceptibility, and health care resource scarcity. The cluster of areas with the highest rates of snakebite risk were seen in the rural and agricultural outskirts of the country. As similarly observed in other parts of the world, areas with the worst snakebite outcome are those with the underserved populations in terms of access to healthcare and infrastructure [19,20]. This points to the need for ensuring equitable distribution of healthcare resources to address the problem of snakebite, particularly in the most susceptible, and exposed populations in the rural areas of Eswatini. Other socio-economic factors also need to be considered to better understand these variations. Poverty and its subsequent consequences are one of the main drivers of these sub-national variations in susceptibility and healthcare resource availability [19]. For example, the lack of sanitation facilities, use of firewood for cooking and heating as well as the type of dwellings that people live in, all of which are factors that are a function of income, determine the risk of snakebite. This is in addition to what has been observed in previous studies which show that the rural population is at high risk of snakebite due to high exposure levels associated with their lifestyle and occupation [8,18,25]. It is, therefore, recommended that interventions to reduce snakebite in Eswatini focus on those geographic areas or clusters that have been shown to have high susceptibility and high healthcare resource scarcity.

We describe the snakebite data collected from the sixteen hospitals and health centres across Eswatini serving as referral snakebite centres. Data from rural and community clinics is excluded as these clinics refer patients with a systemic envenoming to one of the sixteen included hospitals and health centres and these referrals cannot be identified based on the programmatically collected and anonymized data. The number of snakebites without signs of envenoming in our study is therefore an underestimate. We preferred this over distorted numbers of patients with systemic envenoming being counted twice after being referred from these rural and community clinics. In November 2022, snakebite became a notifiable condition in Eswatini, requiring every type of health facility, irrespective of ability to treat snakebite, to report snakebite cases. This move will provide Eswatini with a more accurate picture of the national snakebite burden presenting to the health system in the future. Additionally, community surveys should be considered to better elucidate the snakebite burden in Eswatini, as has been studied in other countries [6,10].

The 2020–2021 snakebite season overlapped with the COVID-19 pandemic. Increased demand for hospital beds and concerns of acquiring COVID-19 may have led to fewer presentations of snakebite to hospitals and health centres]. Finally, to comprehensively assess the snakebite burden in Eswatini, future studies should also include collecting data from animals affected by snakebites.

## Conclusions

Snakebite is a challenging medical and societal issue, affecting all areas and populations in Eswatini. Two cluster risk maps were developed based on risk of snakebite and risk of poor outcomes from a snakebite. Whilst Eswatini is geographically relatively well covered with rural clinics, only hospitals and health centres have the equipment and staffing to safely treat a venomous snakebite with antivenom. Consequently, large areas of the country are at the highest risk of snakebite with poor outcomes. If Eswatini is to meet the global goal of reducing snakebite morbidity and mortality by 50% by 2030, especially in the backdrop of severe shortages of antivenom in the region, the high-risk areas identified in these risk maps are recommended priority areas for resource allocation and targeted snakebite prevention programmes. These risk maps also highlight the urgent need for better, simpler snakebite prevention and treatment tools that can be employed by less skilled health care workers stationed at lower-level community rural clinics.

## Supporting information

S1 Fig Average temperature and rainfall for each region of Eswatini during October 2019 to September 2019 (data from Eswatini Meteorological Service). Made with Natural Earth. Free vector and raster map data @naturalearthdata.com.
(TIF)

S2 Fig Time patients bitten by snakebite, between October 2019 to September 2021, in Eswatini. (TIFF)

S3 Fig Types of adverse reactions to polyvalent snake antivenom recorded from 70 patients. (TIFF)

S4 Fig Venomous snake species richness in Eswatini. Made with shape files from the Eswatini Central Statistics Office (with permission).
(TIF)

S1 Table Datasets used in the snakebite risk analysis. (DOCX)

S2 Table First aid snakebite management implemented by patients. (DOCX)
