# Supplementary figures and images for: Snakebite epidemiology, outcomes and multi-cluster risk modelling in Eswatini
Source: PLoS Negl Trop Dis. 2023 Nov 10;17(11):e0011732. doi: 10.1371/journal.pntd.0011732 (PMC10664941; doi:10.1371/journal.pntd.0011732)

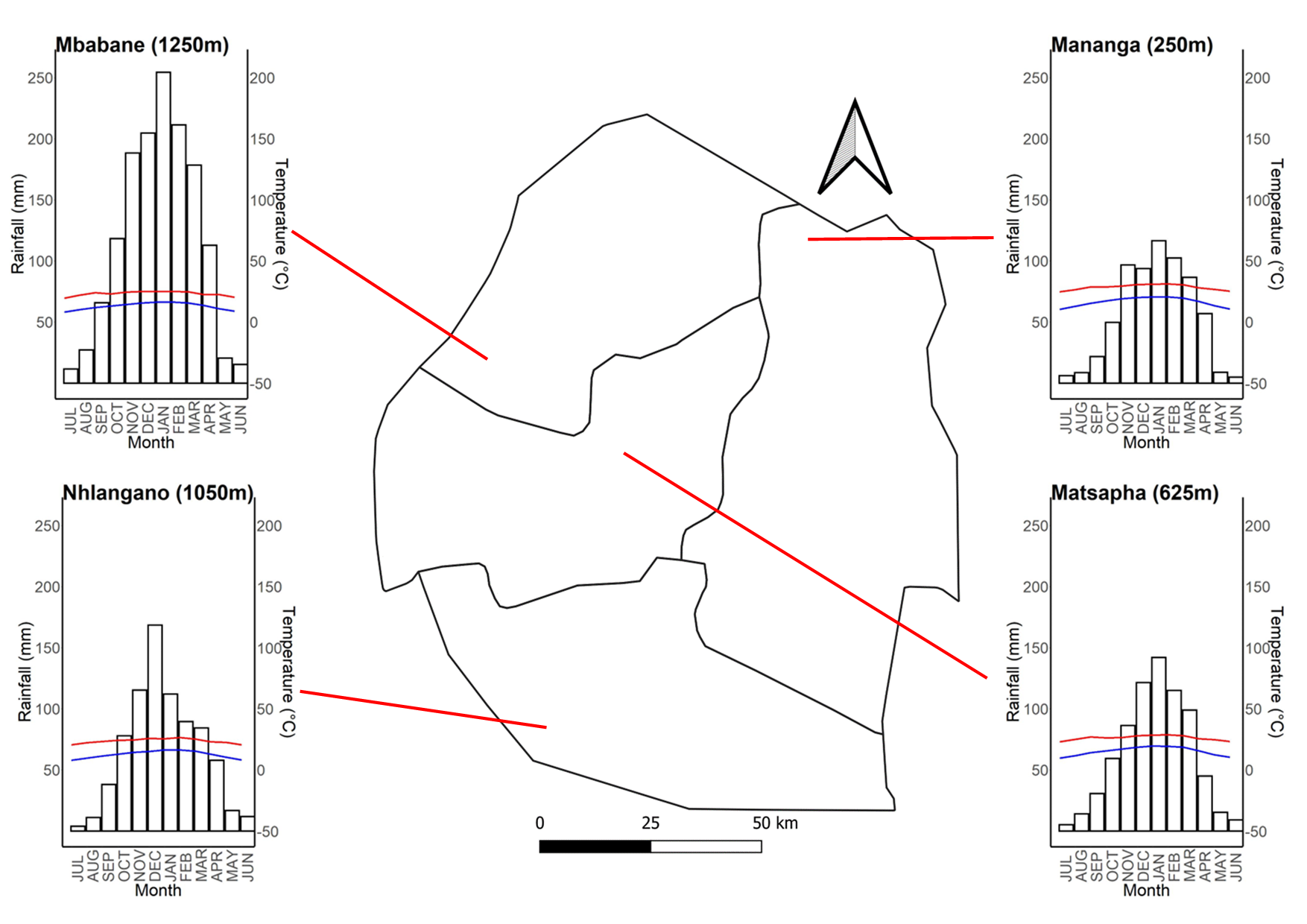

Supplement: S1 Fig — Made with Natural Earth. Free vector and raster map data @naturalearthdata.com. (TIF) [file pntd.0011732.s001.tif]

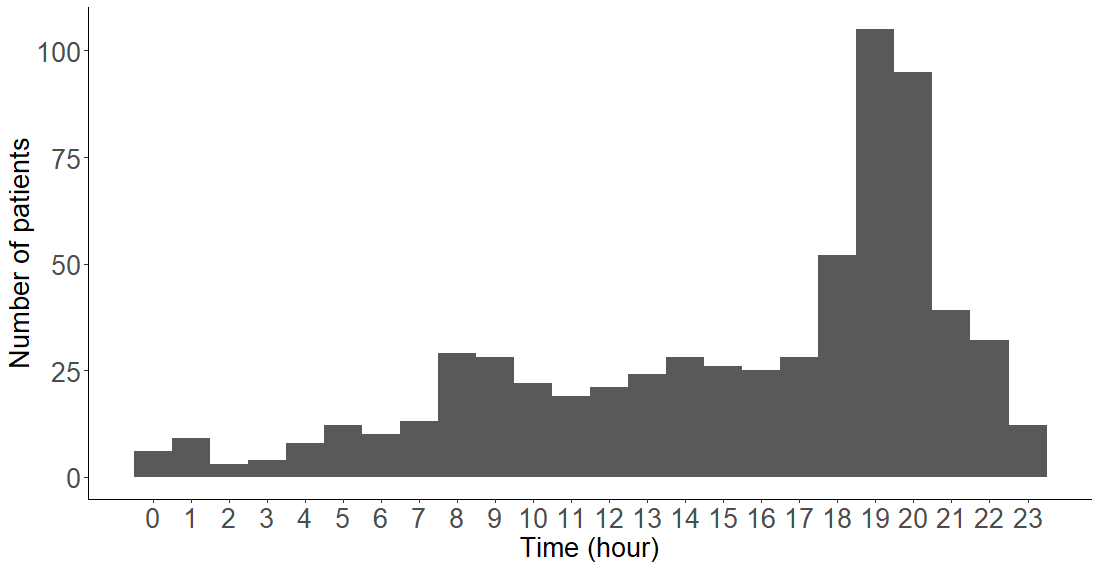

Supplement: S2 Fig — (TIFF) [file pntd.0011732.s002.tiff]

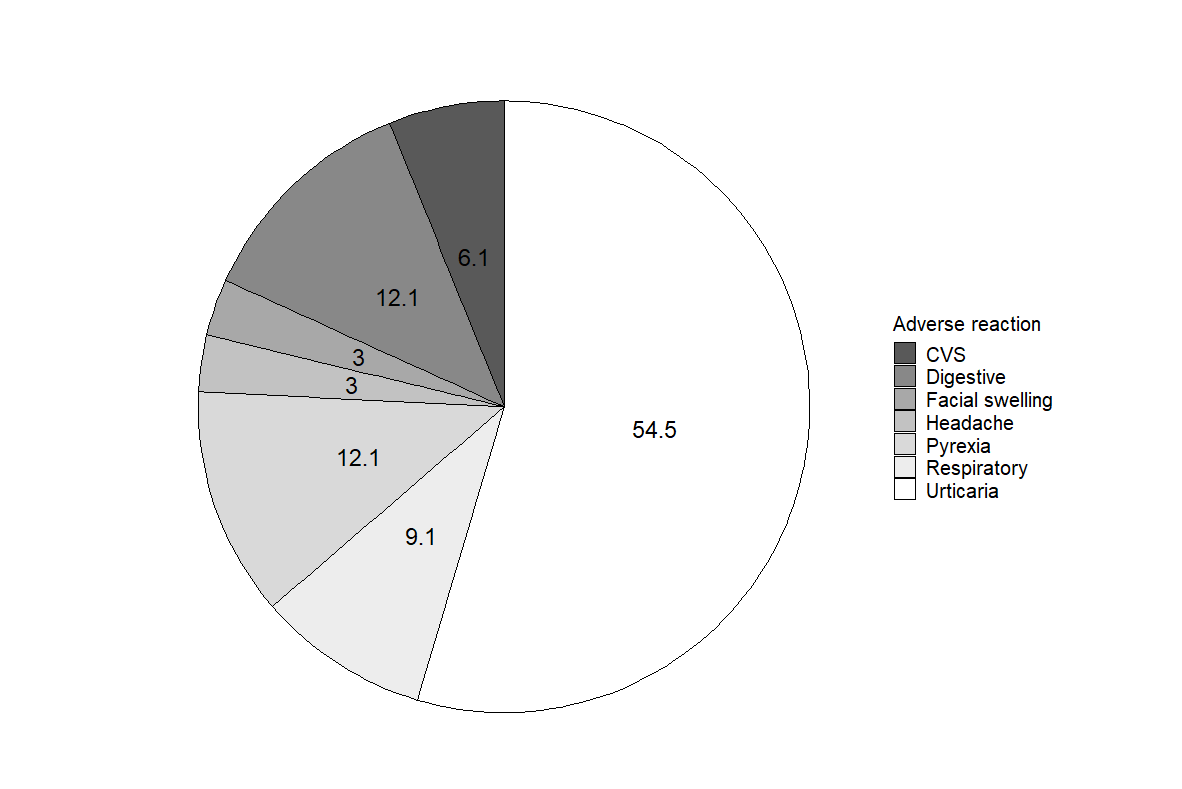

Supplement: S3 Fig — (TIFF) [file pntd.0011732.s003.tiff]

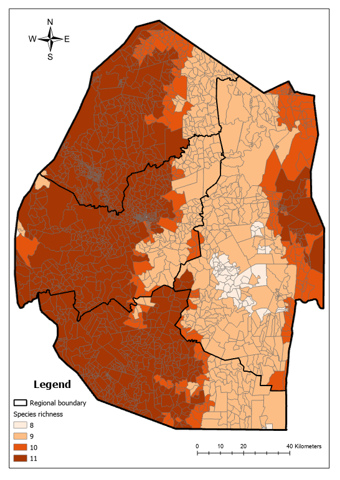

Supplement: S4 Fig — Made with shape files from the Eswatini Central Statistics Office (with permission). (TIF) [file pntd.0011732.s004.tif]
