# Supplementary material for: Snakebite epidemiology, outcomes and multi-cluster risk modelling in Eswatini
Source: PLoS Negl Trop Dis. 2023 Nov 10;17(11):e0011732. doi: 10.1371/journal.pntd.0011732 (PMC10664941; doi:10.1371/journal.pntd.0011732)
Supplement: S1 Table — (DOCX) [file pntd.0011732.s005.docx]

S1 Table: Datasets used in the snakebite risk analysis

| **Dataset** | **Definition** | **Data source** | **Hazard** | **Exposure** | **Susceptibility** | **Healthcare resource scarcity** |
| --- | --- | --- | --- | --- | --- | --- |
| Snakebite incidents | Reported incidents of snakebites | Neglected Tropical diseases Programme, Ministry of Health. | √ |  |  |  |
| Venomous snake species richness map | The species richness of snakes considered to be venomous within Eswatini | Modelled from the occurrence data of the 11 species (see text for further information). | √ |  |  |  |
| Building density | Number of building footprints per unit area (km^2^) | Derived from Sirko et al. [43] |  | √ |  |  |
| Average number of occupants in a building | The average number of occupants in a building | Derived from population and building footprint data |  | √ |  |  |
| Poultry density | Density of poultry owned by households | The 2017 Population and Housing Census [44] |  | √ |  |  |
| Proportion of tree cover | Percentage of land areas under tree cover | Derived from Brown et al [45] |  | √ |  |  |
| Proportion of cropland | Percentage of land area under cropland | Derived from Brown et al [45] |  | √ |  |  |
| Proportion of built-up areas | Percentage of land area under built-up area | Derived from Brown et al [45] |  | √ |  |  |
| Land cover edge density | The total length of land cover edge divided by the land area (k/km^2^) | Derived from Brown et al [45] |  | √ |  |  |
| Land cover Shannon diversity | Shannon's diversity index as a measure of the variety of land cover types in an area. | Derived from Brown et al [45] |  | √ |  |  |
| Poverty headcount | Percentage of households living below USD 2 per day poverty line | Eswatini Household Income and Expenditure Survey [46] |  |  | √ |  |
| Households use of firewood for cooking | The percentage of households that use firewood for cooking | The 2017 Population and Housing Census [44] |  |  | √ |  |
| Households use of paraffin for lighting | The percentage of households that use paraffin for lighting | The 2017 Population and Housing Census [44] |  |  | √ |  |
| Households using candles for lighting | The percentage of households that use candles for lighting | The 2017 Population and Housing Census [44] |  |  | √ |  |
| Proportion of youth and children | The percentage of the population that is below the age of 44 | The 2017 Population and Housing Census [44] |  |  | √ |  |
| Proportion of elderly | The percentage of the population that is above the age of 60 | The 2017 Population and Housing Census [44] |  |  | √ |  |
| Employment rate | Percentage of employed population that is employed in outdoor/field environments | The 2017 Population and Housing Census [44] |  |  | √ |  |
| Households access to sanitation facilities | Percentage of households that have access to sanitation facilities like flushing toilets and tap water. | The 2017 Population and Housing Census [44] |  |  | √ |  |
| Travel time to the nearest healthcare facility | Distance to the nearest healthcare facility that can treat snakebites (minutes) | Estimated from Ministry of Health’s healthcare facility data |  |  |  | √ |
