# Supplementary material for: Snakebite epidemiology, outcomes and multi-cluster risk modelling in Eswatini
Source: PLoS Negl Trop Dis. 2023 Nov 10;17(11):e0011732. doi: 10.1371/journal.pntd.0011732 (PMC10664941; doi:10.1371/journal.pntd.0011732)
Supplement: S2 Table — (DOCX) [file pntd.0011732.s006.docx]

S2 Table: First aid snakebite management implemented by patients

| **First aid applied by patient** | **Total (%)** |
| --- | --- |
| *Yes** | 615 (66.0) |
| Bandage | 16 |
| Herbal remedy applied | 43 |
| Herbal remedy ingested | 138 |
| Incision | 83 |
| Tourniquet | 508 |
| Other | 39 |
| None | 289 (30.7) |
| Not recorded | 31 (3.3) |

*Figures for types of first aid measures total more than 615 because one or more types of first aid treatments were employed by the 615 patients
